# Body shame and disturbed eating behaviors: an ecological momentary assessment approach

**DOI:** 10.1192/j.eurpsy.2022.1493

**Published:** 2022-09-01

**Authors:** D. Nechita

**Affiliations:** Babes-Bolyai University Cluj-Napoca , Department Of Clinical Psychology And Psychotherapy, Cluj-Napoca , Romania

**Keywords:** Ecological Momentary Assessment, body shame, Eating Disorders

## Abstract

**Introduction:**

There is a well-established body of investigations showing that the experience of shame is associated with eating disorders symptoms. Meta-analytical data indicate that body shame is one type of shame that seems to be especially relevant in case of eating disorders. While there are many studies which investigated the association between the predisposition to feel ashamed about one’s body and eating disturbance, there are virtually no inquires on how momentary body shame is related to disturbed eating behaviors.

**Objectives:**

In this study we aimed to investigate the relationship between momentary body shame and disturbed eating behaviors using an intensive longitudinal design.

**Methods:**

Females with high levels of eating disorders symptoms completed five, randomly-initiated surveys per day delivered via a smartphone application for a total of two weeks. The survey evaluated the level of body shame and disturbed eating behaviors (i.e., binge eating, purging, excessive exercises, body checking).

**Results:**

Preliminary results indicate that higher levels of body shame are associated with higher levels of disturbed eating behaviors. The level of body shame was higher in binge eating days compared with non-binge eating days.

**Conclusions:**

Fluctuations in body shame seem to contribute to the maintenance of disturbed eating behaviors. Implications and limitations of these findings are discussed.

**Disclosure:**

No significant relationships.

